# Role of prelimbic cortex PKC and PKMζ in fear memory reconsolidation and persistence following reactivation

**DOI:** 10.1038/s41598-020-60046-x

**Published:** 2020-03-05

**Authors:** Thiago Rodrigues da Silva, Ana Maria Raymundi, Leandro José Bertoglio, Roberto Andreatini, Cristina A. Stern

**Affiliations:** 1 Pharmacology Department, Federal University of Parana, Curitiba, Brazil; 2 Pharmacology Department, Federal University of Santa Catarina, Florianopolis, Brazil

**Keywords:** Prefrontal cortex, Fear conditioning, Long-term memory

## Abstract

The persistence of newly acquired memories is supported by the activity of PKMζ, an atypical isoform of protein kinase C (PKC). Whether the activity of conventional and atypical PKC isoforms contributes to reactivated memories to persist is still unknown. Similarly, whether memory reactivation is a prerequisite for interventions to be able to change memory persistence is scarcely investigated. Based on the above, we examined the role of conventional and atypical PKC isoforms in the prelimbic cortex in reconsolidation and persistence of a reactivated contextual fear memory in male Wistar rats. It is shown that (i) inhibiting the PKC activity with chelerythrine or the PKMζ activity with ZIP impaired the persistence of a reactivated memory for at least 21 days; (ii) ZIP given immediately after memory reactivation affected neither the reconsolidation nor the persistence process. In contrast, when given 1 h later, it impaired the memory persistence; (iii) chelerythrine given immediately after memory reactivation impaired the reconsolidation; (iv) omitting memory reactivation prevented the chelerythrine- and ZIP-induced effects: (v) the ZIP action is independent of the time elapsed between its administration and the initial memory test. The results indicate that prelimbic cortex PKC and PKMζ are involved in memory reconsolidation and persistence.

## Introduction

The family of the protein kinase C (PKC) includes conventional (α, β, and γ), novel (δ, ɛ, η, and θ), and atypical (ζ, ι, and λ) isoforms^1,2^. The role of each one of these PKC isoforms is currently under investigation. Accumulating evidence suggests that persistent activation of an atypical protein kinase C isoform (PKMζ) at the late phase of memory consolidation is responsible for maintaining long-term potentiation and long-term memory^3–5^. PKMζ has also been reported to be necessary for the persistence of newly acquired procedural, spatial, appetitive (rewarding), and aversive memories^4,6–8^. The overexpression of PKMζ in the prelimbic (PL) cortex has been shown to potentiate the aversive memory persistence^9^. On the other hand, its DNA methylation in the PL cortex, or its aggregation with neurofibrillary tangles in the hippocampus, has been associated with memory decline or impairment in animal models of aging-related diseases^10,11^.

It has been proposed that a brief memory reactivation induces a late phase (beyond the reconsolidation time-window) of protein synthesis that might underlie the persistence of reactivated aversive memories^12^. Accordingly, a protein synthesis inhibitor given 9.5 h after reactivation has been reported to impair the persistence of a reactivated fear memory^13^. Similarly, PKC inhibition 6 or 9 h after fear memory reactivation has impaired its persistence without affecting the reconsolidation process^14^. There is some similarity between fear memory consolidation and reconsolidation, such as their time-window of occurrence^15–17^. The mechanisms supporting memory persistence during and after memory consolidation are relatively more investigated^18,19^. The investigation of memory maintenance mechanisms during and after the reconsolidation time-window is still incipient. It would be of scientific and therapeutic relevance to advance our knowledge about the latter question.

The PL cortex has been implicated in aversive memory reconsolidation^20,21^. PKMζ expression in this medial prefrontal cortex sub-region also seems to support the maintenance of newly acquired aversive memories^22^. Based on these facts, we hypothesized that not only PKC but also the PKMζ isoform could support the persistence of a reactivated aversive memory. Therefore, the main objective of the present work was to investigate the role of PL cortex PKC and PKMζ in the persistence of contextual fear memory in rats. Initially, we focused on specific time points after the end of the memory reconsolidation time-window (from 6 to 18 h after memory reactivation). Next, by omitting memory reactivation, we assessed the requirement of this process to induce memory persistence mechanisms. Finally, we addressed whether PKC and/or PKMζ is also important for memory reconsolidation and/or persistence within the reconsolidation time-window (0 and/or 1 h after memory reactivation).

## Results

### Effects of PL cortex PKC inhibition by chelerythrine on the persistence of a reactivated contextual fear memory

**Figure 1:**
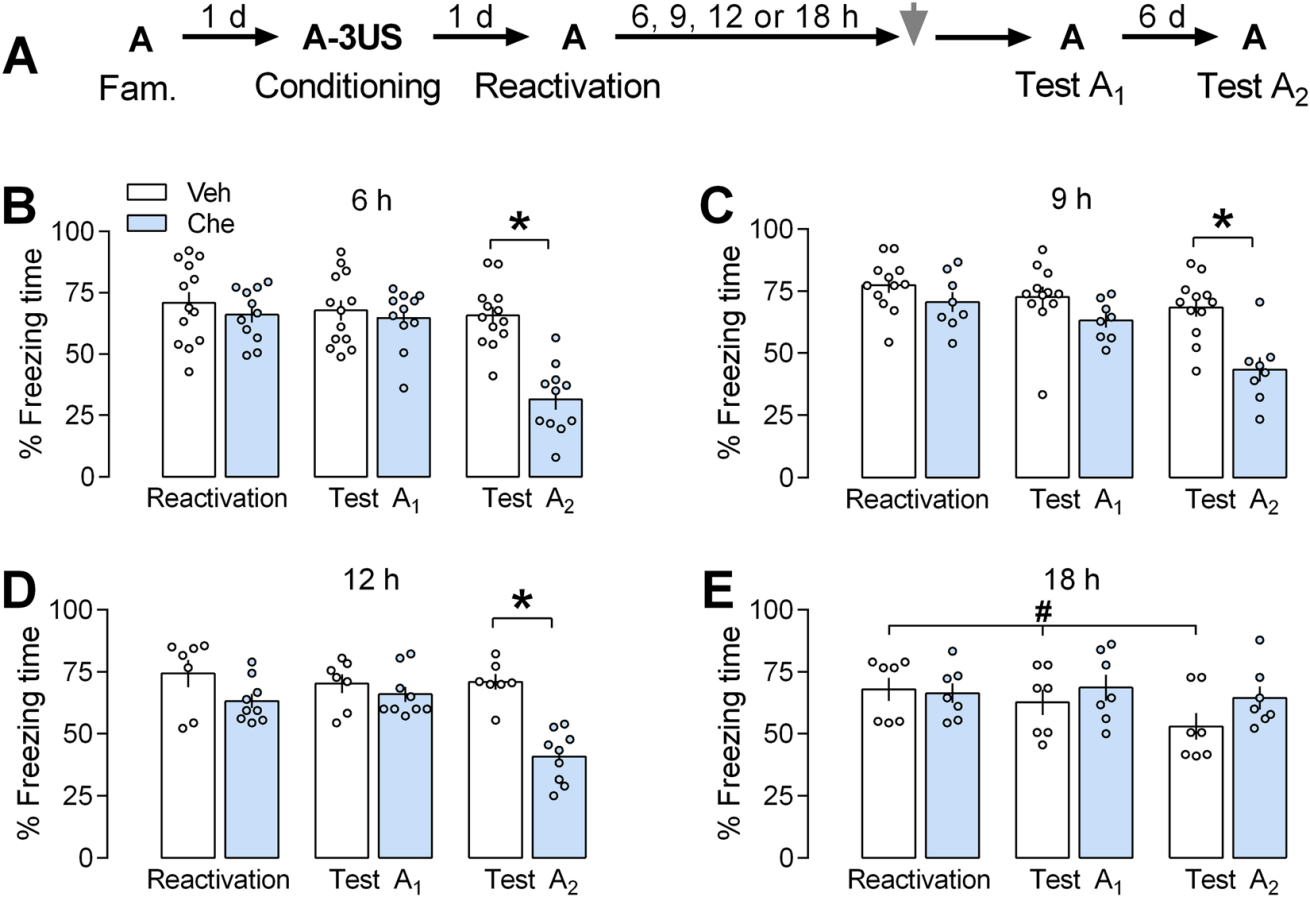
Effects of prelimbic (PL) cortex PKC inhibition by chelerythrine (Che) on the persistence of a reactivated contextual fear memory. **(A)** The general experimental design used. Animals were initially familiarized to Context A. A day later, the context was paired with three shocks (US). On the next day, from 6 to 18 h after memory reactivation (Context A re-exposure), independent groups of animals received a bilateral infusion of vehicle (Veh) or Che (3.0 nmol) intra-PL cortex. One and seven days later, the animals were re-exposed to the paired context (Tests A_1_ and A_2_) to assess the Che effects on memory. (**B)** Effects of Che on memory persistence when given 6 h after reactivation. Che-treated animals presented less freezing time than controls during Test A_2,_ suggesting an impairment of memory persistence. (**C)** Che effects on memory persistence when given 9 h after reactivation. Che-treated animals presented less freezing time than controls during Test A_2,_ suggesting an impairment of memory persistence. (**D)** Che effects on memory persistence when given 12 h after reactivation. Che-treated animals presented less freezing time than controls during Test A_2,_ suggesting an impairment of memory persistence. (**E)** Che effects on memory persistence when given 18 h after reactivation. Che-treated animals presented freezing time similar to controls during Test A_2,_ suggesting no memory persistence changes. Values are expressed as mean ± S.E.M (number of animals per group: B = 11–13; C = 8–12; D = 7–9; E = 7/group). The asterisk denotes a statistically significant difference (*P* < 0.05) from the respective control group. The fence (hashtag) denotes a statistically significant difference (*P* < 0.05) from the control group during memory reactivation (mixed ANOVA followed by the Tukey test).

We tested this pharmacological intervention 6, 9, 12, and 18 h after memory reactivation (Fig. 1A). At the first time point selected, a mixed ANOVA showed significant effects of the sessions [F_(2,44)_ = 33.7; *P* < 0.0001], the treatment [F_(1,22)_ = 8.77; *P* = 0.007], and the interaction between these factors [F_(2,44)_ = 22.0; *P* = 0.00001], for freezing time. As shown in Fig. 1B, the Tukey *post-hoc* test showed a significant difference between control and chelerythrine groups (n = 13 and 11, respectively) during Test A_2_ (*P* = 0.0001; Hedges’ *g* effect size = 2.65), but not the reactivation session (*P* = 0.90; *g* = 0.40) or Test A_1_ (*P* = 0.95; *g* = 0.34).

At the second time point selected (9 h), a mixed ANOVA showed significant effects of the sessions [F_(2,36)_ = 25.1; *P* < 0.0001], the treatment [F_(1,18)_ = 8.95; *P* = 0.0078], and their interaction [F_(2,36)_ = 7.06; *P* = 0.0026]. As shown in Fig. 1C, there was a significant difference between control and chelerythrine groups (n = 12 and 8, respectively) during Test A_2_ (*P* = 0.001; *g* = 1.92), but not the reactivation session (*P* = 0.81; *g* = 0.63) or Test A_1_ (*P* = 0.53; *g* = 0.77).

At the third time point selected (12 h), a mixed ANOVA showed significant effect of the sessions [F_(2,28)_ = 20.9; *P* < 0.0001], the treatment [F_(1,14)_ = 11.3; *P* = 0.0047], and their interaction [F_(2,28)_ = 18.0; *P* < 0.0001]. As shown in Fig. 1D, there was a significant difference between control and chelerythrine groups (n = 7 and 9, respectively) during Test A_2_ (*P* = 0.0002; *g* = 3.15), but not the reactivation session (*P* = 0.31; *g* = 0.75) or Test A_1_ (*P* = 0.96; *g* = 0.43).

At the fourth time point selected (18 h), a mixed ANOVA showed significant effects of the sessions [F_(2,24)_ = 9.43; *P* < 0.0001], and an interaction between sessions and treatment [F_(2,24)_ = 5.00; *P* = 0.02], but not the treatment [F_(1,12)_ = 0.66; *P* = 0.43]. As shown in Fig. 1E, there was a significant difference between Test A_2_ and the reactivation session in animals treated with vehicle (*P* = 0.03; *g* = 1.12; n = 7), but not chelerythrine (*P* = 0.57; *g* = 0.76; n = 7). There were no significant differences between groups during any session performed.

Altogether, the results associate the PL cortex PKC activity 6, 9 or 12 h after reactivating an aversive memory with its persistence.

### Impaired memory persistence induced by PKC inhibition in the PL cortex requires prior memory reactivation

**Figure 2:**
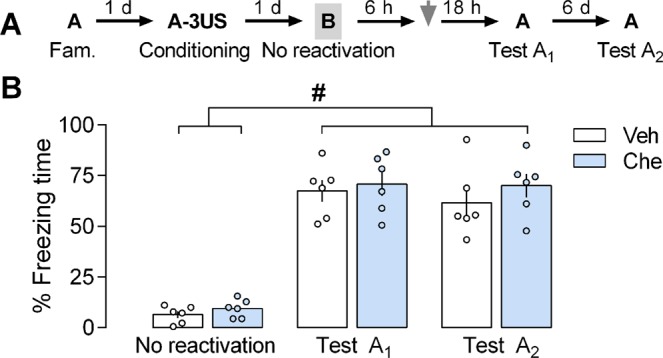
Effects of prelimbic (PL) cortex PKC inhibition by chelerythrine (Che) on the contextual fear memory persistence without prior reactivation. **(A)** The general experimental design used. Animals were initially familiarized to Context A. A day later, the context was paired with three shocks (US). On the next day, 6 h after omitting memory reactivation (neutral and unpaired Context B exposure), the animals received a bilateral infusion of vehicle (Veh) or Che (3.0 nmol) intra-PL cortex. One and seven days later, the animals were re-exposed to the paired context (Tests A_1_ and A_2_) to assess the Che effects on memory. **(B)** Che effects on memory persistence when given 6 h after omitting memory reactivation. Che-treated animals presented freezing time similar to controls during Test A_2,_ suggesting no changes in memory persistence. Values are expressed as mean ± S.E.M (6/group). The fence (hashtag) denotes a statistically significant difference (*P* < 0.05) from Tests A_1_ and A_2_ relative to the no reactivation session in both groups (mixed ANOVA followed by the Tukey test).

Changes in memory persistence induced by chelerythrine were similar when this drug was given 6, 9 or 12 h after the reactivation session. Based on this, the first time point was selected to conduct the next experiment in which the session of memory reactivation was omitted (Fig. 2A) to investigate whether it is a prerequisite for the observation of the above-mentioned outcome. To this aim, contextually fear-conditioned animals were exposed to a neutral and unpaired Context B (the no reactivation session) and 6 h later treated with vehicle or chelerythrine (n = 6 per group). A mixed ANOVA showed significant effects of the sessions [F_(2,20)_ = 167,48; *P* = 0.000001], but not the treatment [F_(1,10)_ = 0.75; *P* = 0.40] or their interaction [F_(2.20)_ = 0.34; *P* = 0.71]. As shown in Fig. 2B, both groups presented higher freezing values during Test A_1_ and A_2_ than in the no reactivation session (P < 0.0001), confirming that this conditioned behavior is observed predominantly when the animals are re-exposed to the conditioning context. Further, no treatment effect was observed, indicating that the memory reactivation is essential for chelerythrine-induced impairments in its persistence.

### Effects of PL cortex PKMζ inhibition by ZIP on the persistence of a reactivated contextual fear memory

**Figure 3:**
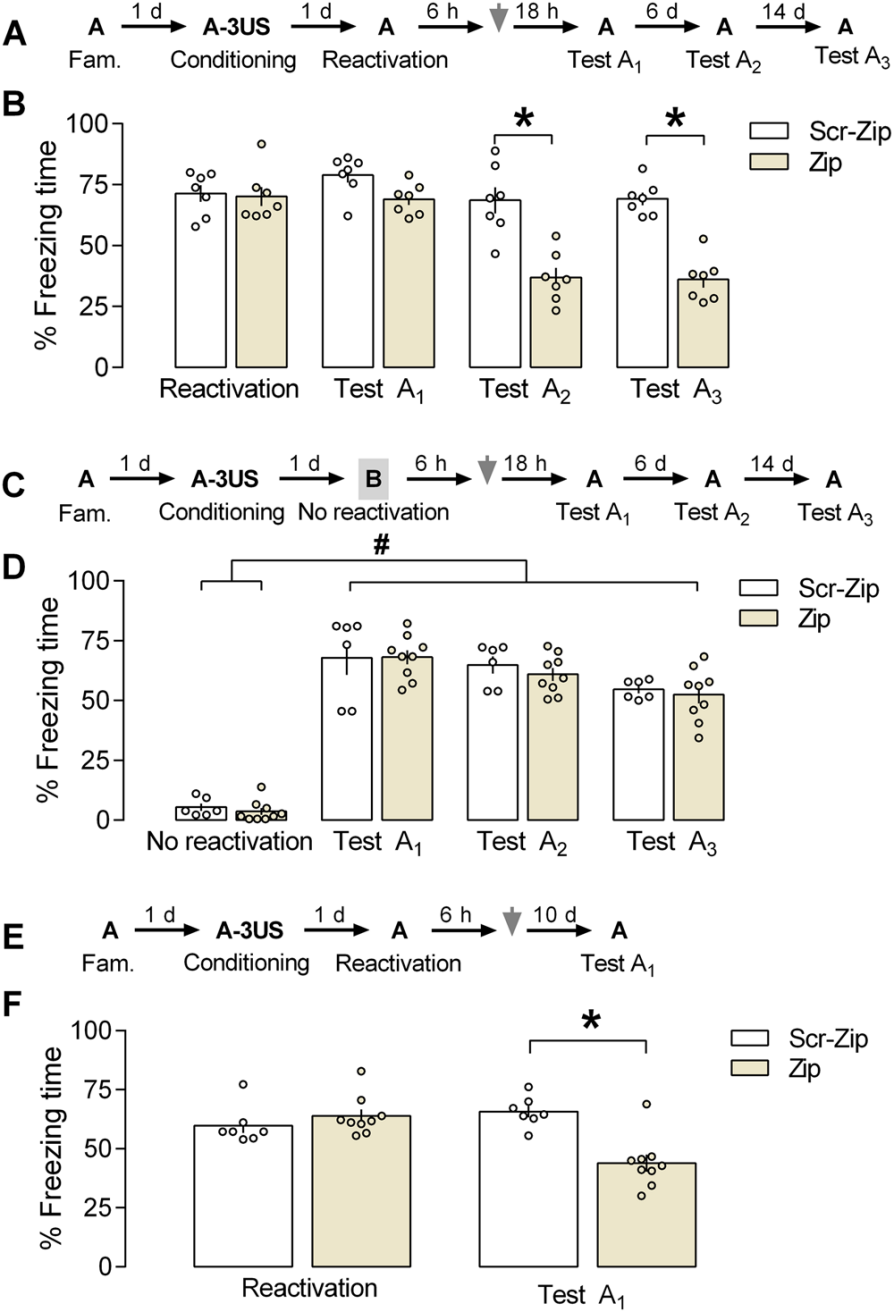
Effects of prelimbic (PL) cortex PKMζ inhibition by ZIP on the persistence of a reactivated contextual fear memory. **(A)** The general experimental design used. Animals were initially familiarized to Context A. A day later, the context was paired with three shocks (US). On the next day, 6 h after memory reactivation (Context A re-exposure), the animals received a bilateral infusion of ZIP or Scr-ZIP (10 nmol) intra-PL cortex. One, seven and 21 days later, the animals were re-exposed to pairing context (Tests A_1,_ A_2_, and A_3_) to assess the ZIP effects on memory. (**B)** Effects of ZIP on memory persistence when given 6 h after reactivation. ZIP-treated animals presented less freezing time than controls during Tests A_2_ and A_3,_ suggesting an impairment in memory persistence. **(C)** The general experimental design used. Animals were initially familiarized to Context A. A day later, the context was paired with three shocks (US). On the next day, 6 h after omitting memory reactivation (neutral and unpaired Context B exposure), the animals received a bilateral infusion of ZIP or Scr-ZIP intra-PL cortex. One, seven and 21 days later, the animals were re-exposed to the paired context (Tests A_1,_ A_2_, and A_3_) to assess ZIP effects on memory. **(D)** ZIP effects on memory persistence when given 6 h after omitting memory reactivation. ZIP-treated animals presented freezing time similar to controls during any test_,_ suggesting no changes in memory persistence. **(E)** The general experimental design used. Animals were initially familiarized to Context A. A day later, the context was paired with three shocks (US). On the next day, 6 h after memory reactivation (Context A re-exposure), the animals received a bilateral infusion of ZIP or Scr-ZIP intra-PL cortex. Ten days later, the animals were re-exposed to the paired context (Test A_1_) to assess ZIP effects on memory. (**F)** Effects of ZIP on memory persistence when given 6 h after reactivation. ZIP-treated animals presented less freezing time than controls during Test A_1_, suggesting an impairment in memory persistence. Values are expressed as mean ± S.E.M (number of animals per group: B = 7/group; D = 6–9; F = 7–9). In “B” and “F”, the asterisk denotes a statistically significant difference (*P* < 0.05) from the respective control group (mixed ANOVA followed by the Tukey test). In “D”, the fence (hashtag) denotes a statistically significant difference (*P* < 0.05) from Tests A_1_, A_2_ and A_3_ relative to the no reactivation session in both groups (mixed ANOVA followed by the Tukey test).

The overexpression of PKMζ in the PL cortex has been shown to potentiate the aversive memory persistence^9^. Based on this and preceding chelerythrine findings, we investigated whether PKMζ activity in the PL cortex 6 h after reactivating a contextual fear memory is involved in its persistence (Fig. 3A). A mixed ANOVA showed significant effects of the sessions [F_(3,36)_ = 31.4; *P* < 0.0001], the treatment [F_(1,12)_ = 25.9; *P* = 0.0003], and their interaction [F_(3,36)_ = 15.1; *P* = 0.0001]. As shown in Fig. 3B, there were significant differences between control and ZIP groups (n = 7 in both cases) during Test A_2_ (*P* = 0.0001; *g* = 2.53), and Test A_3_ (*P* = 0.0001; *g* = 4.11), but not the reactivation session (*P* = 0.99; *g* = 0.13) or Test A_1_ (*P* = 0.54; *g* = 1.35). Altogether, the results associate the PL cortex PKMζ activity 6 h after reactivating an aversive memory with its persistence.

To investigate whether memory reactivation is a prerequisite for ZIP to affect the process of persistence, in the next experiment animals were exposed to a neutral and unpaired Context B (the no reactivation session) and 6 h later treated with vehicle (n = 6) or ZIP (n = 9) (Fig. 3C). A mixed ANOVA showed significant effects of the sessions [F_(3,39)_ = 202; *P* = 0.00001], but not the treatment [F_(1,13)_ = 0.33; *P* = 0.57] or their interaction [F_(3,39)_ = 0.17; *P* = 0.91]. As shown in Fig. 3D, both groups presented higher freezing time during Tests A_1,_ A_2_ and A_3_ than in the no reactivation session (*P* < 0.0001), confirming that fear expression is more selective to the conditioning context. Moreover, no treatment effect was observed,_,_ indicating that memory reactivation is necessary for the ZIP-induced changes in the memory persistence.

It has been argued that inhibiting PKM*ζ* may impair memory expression rather than permanently interfering with its persistence^23^. To investigate whether drug-induced effects on persistence of a reactivated memory depends on the time elapsed between treatment and Test A_1_ (Fig. 3E), in the next experiment the animals received treatment infusion into the PL cortex 6 h after memory reactivation and Test A_1_ was conducted after 10 days (instead of 1 day later). A mixed ANOVA showed significant effects of the sessions [F_(1,14)_ = 5.68; *P* < 0.03], the treatment [F_(1,14)_ = 7.40; *P* = 0.01], and their interaction [F_(1,14)_ = 19.41; *P* = 0.0006]. As shown in Fig. 3F, there was a significant difference between control and ZIP groups (n = 7–9 animals/group) during Test A_1_ (*P* = 0.01; *g* = 2.37), but not the reactivation session (*P* = 0.78; *g* = 0.50). These results corroborate that PL cortex PKMζ activity 6 h after reactivating a contextual fear memory is involved in its persistence, and indicate the ZIP action is independent of the time elapsed between its administration and Test A_1_.

### Effects of PL cortex PKC inhibition by chelerythrine on the reconsolidation of a reactivated contextual fear memory

**Figure 4:**
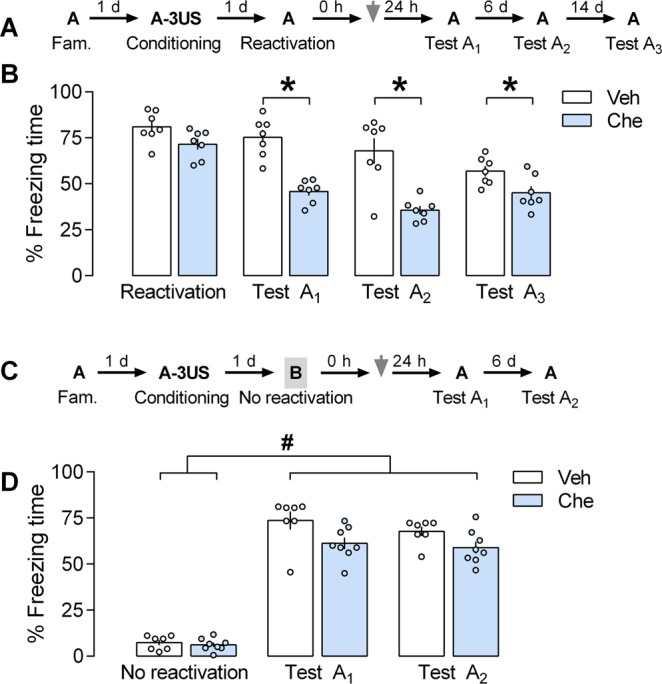
Effects of prelimbic (PL) cortex PKC inhibition by chelerythrine (Che) on the reconsolidation of a reactivated contextual fear memory **(A)** The general experimental design used. Animals were initially familiarized to Context A. A day later, the context was paired with three shocks (US). On the next day, immediately after memory reactivation (Context A re-exposure), the animals received a bilateral infusion of vehicle (Veh) or Che (3.0 nmol) intra-PL cortex. One, seven and 21 days later, the animals were re-exposed to the paired context (Tests A_1,_ A_2_ and A_3_) to assess the Che effects on memory. (**B)** Effects of Che on memory reconsolidation when given immediately after reactivation. Che-treated animals presented less freezing time than controls during Tests A_1,_ A_2_ and A_3,_ suggesting an impairment in memory reconsolidation. **(C)** The general experimental design used. Animals were initially familiarized to the Context A. A day later, the context was paired with three shocks (US). On the next day, immediately after omitting memory reactivation (neutral and unpaired Context B exposure), the animals received a bilateral infusion of Veh or Che intra-PL cortex. One and seven days later, the animals were re-exposed to the paired context (Tests A_1_ and A_2_) to assess Che effects on memory. **(D)** Che effects on memory reconsolidation when given immediately after omitting memory reactivation. Che-treated animals presented freezing time similar to controls during Test A_2,_ suggesting no changes in memory reconsolidation. Values are expressed as mean ± S.E.M (number of animals per group: B = 7/group; D = 6/group). In “B”, the asterisk denotes a statistically significant difference (*P < 0.05) from the respective control group (mixed-ANOVA followed by the Tukey test). In “D”, the fence (hashtag) denotes a statistically significant difference (*P* < 0.05) from Tests A_1_ and A_2_ relative to the no reactivation session in both groups (mixed ANOVA followed by the Tukey test).

We tested this pharmacological intervention immediately after the memory reactivation session (Fig. 4A). A mixed ANOVA showed significant effects of the sessions [F_(3,36)_ = 22.5; *P* < 0.0001], the treatment [F_(1,12)_ = 40.8; *P* = 0.0001], and their interaction [F_(3,36)_ = 5.68; *P* = 0.0027]. As shown in Fig. 4B, there were significant differences between control and chelerythrine groups (n = 7 in both cases) during Test A_1_ (*P* = 0.0003; *g* = 2.95), Test A_2_ (*P* = 0.0001; *g* = 4.30), and Test A_3_ (*P* = 0.003; *g* = 1.26), but not the reactivation session (*P* = 0.60; *g* = 0.79). These results indicate that PL cortex PKC activity immediately after reactivating a contextual fear memory is involved in its reconsolidation, and the drug-induced reconsolidation impairment was still present 21 days later.

To investigate whether memory reactivation is a prerequisite for chelerythrine to affect the reconsolidation process, in the next experiment the reactivation session was omitted because the animals were exposed to context B and then treated with vehicle or chelerythrine (Fig. 4C; n = 6/group). A mixed ANOVA showed significant effects of the sessions [F_(2,26)_ = 379.7; *P* = 0.0001], but not the treatment [F_(1,13)_ = 5.71; *P* = 0.06], or their interaction [F_(2, 26)_ = 2.76; *P* = 0.09]. As shown in Fig. 4D, both groups presented higher freezing time during Tests A_1_ and A_2_ than in the no reactivation session (*P* < 0.0001), confirming that fear expression is more selective to the conditioning context. Moreover, there was no treatment effect, indicating that memory reactivation is also necessary for drug-induced effects in memory reconsolidation.

### Effects of PL cortex PKMζ inhibition by ZIP on the reconsolidation of a reactivated contextual fear memory

**Figure 5:**
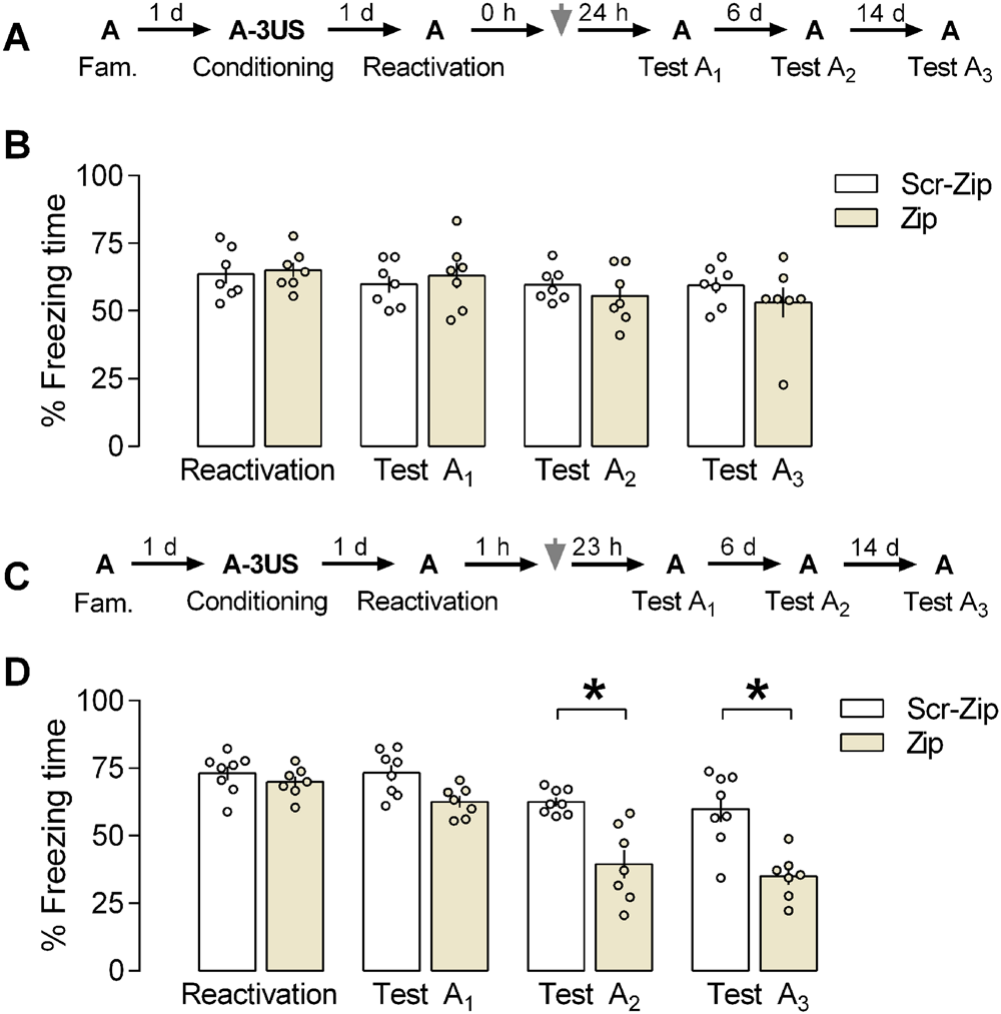
Effects of prelimbic (PL) cortex PKMζ inhibition by ZIP on the reconsolidation of a reactivated contextual fear memory. **(A)** The general experimental design used. Animals were initially familiarized to Context A. A day later, the context was paired with three shocks (US). On the next day, immediately after memory reactivation (Context A re-exposure), the animals received a bilateral infusion of ZIP or Scr-ZIP (10 nmol) intra-PL cortex. One, seven and 21 days later, the animals were re-exposed to the paired context (Tests A_1,_ A_2_, and A_3_) to assess ZIP effects on memory. **(B)** ZIP effects on memory reconsolidation when given immediately after memory reactivation. ZIP-treated animals presented freezing time similar to controls during Tests A_1_, A_2_ and A_3,_ suggesting no changes in memory reconsolidation. **(C)** The general experimental design used. Animals were initially familiarized to Context A. A day later, the context was paired with three shocks (US). On the next day, 1 h after memory reactivation (Context A re-exposure), the animals received a bilateral infusion of ZIP or Scr-ZIP intra-PL cortex. One, seven and 21 days later, the animals were re-exposed to the paired context (Tests A_1,_ A_2_, and A_3_) to assess ZIP effects on memory. **(D)** ZIP effects on memory persistence when given 1 h after reactivation. ZIP-treated animals presented less freezing time than controls during Tests A_2_ and A_3,_ suggesting an impairment in memory persistence. Values are expressed as mean ± S.E.M (number of animals per group: B = 7/group; D = 7–8). In “B” the mixed ANOVA followed by Tukey test, showed no significant difference during paired context exposures (context A). In “D”, the asterisk denotes a statistically significant difference (*P* < 0.05) from the respective control group (mixed ANOVA followed by the Tukey test).

We tested this pharmacological intervention immediately after the memory reactivation session and 1 h later. At the first time point selected (Fig. 5A), a mixed ANOVA showed no significant effects of the sessions [F_(3,36)_ = 4.33; *P* < 0.303], the treatment [F_(1,12)_ = 0.109; *P* = 0.7472], and their interaction [F_(3,36)_ = 1.62; *P* = 0.19]. As shown in Fig. 5B, there were no significant differences between control and ZIP groups (n = 7 in both cases) during any session performed, suggesting that PKMζ activity in the PL cortex immediately after reactivating a contextual fear memory is not involved in its reconsolidation (and persistence).

At the second time point selected (Fig. 5C), a mixed ANOVA showed significant effects of the sessions [F_(3,39)_ = 42.2; *P* < 0.0001], the treatment [F_(1,13)_ = 20.2; *P* = 0.0006], and their interaction [F_(3,39)_ = 7.82; *P* = 0.0003]. As shown in Fig. 5D, there were significant differences between control and ZIP groups (n = 8 and 7, respectively) during Test A_2_ (*P* = 0.0005; *g* = 2.25), and Test A_3_ (*P* = 0.0002; *g* = 2.19), but not the reactivation session (*P* = 0.99; *g* = 0.48) or Test A_1_ (*P* = 0.35; *g* = 1.48). Altogether, the results associate the PL cortex PKMζ activity as early as 1 h after reactivating an aversive memory with its persistence.

## Discussion

The present study sought to investigate the role of PL cortex PKC and PKMζ in the reconsolidation and persistence of a reactivated contextual fear memory in rats. It was shown that: 1) activity of both PKC and PKMζ are necessary for the persistence of a reactivated memory; 2) effects of PKC or PKMζ inhibition require prior memory reactivation; 3) effects of PKMζ inhibition are independent of the initial test 1 day after ZIP infusion; 4) activity of PKC, but not PKMζ, is also involved in reconsolidation of a reactivated memory; 5) activity of PKMζ during the reconsolidation time-window is important for memory persistence. These findings indicate that PL cortex PKC and PKMζ have a differential involvement in the processes examined.

The chelerythrine-induced PKC inhibition 6 h after memory reactivation had no effects on freezing time when the animals were tested one day later (Test A_1_). This result corroborates prior evidence showing that various experimental interventions delivered at this time point are no longer able to interfere with aversive memory reconsolidation^15,16,24^. However, after seven days (Test A_2_) there was a difference between groups, suggesting that PKC activity in the PL cortex is required for the reactivated aversive memory to persist. This pattern of results (reduced freezing time 7 days but not 24 h after drug treatment) is in line with those reported after infusing a protein synthesis inhibitor into the basolateral amygdala^13^, an ERK pathway inhibitor into the dorsal hippocampus^25^, and a systemic administration of a non-selective PKC inhibitor^14^.

The processes associated with aversive memory persistence, such as the second wave of Arc protein expression in the basolateral amygdala^26^, have commonly been reported to occur up to 12 h after its reactivation. To further investigate whether PKC activity in the PL cortex is important for memory persistence, the chelerythrine effects were assessed in independent groups of animals 9, 12 or 18 h post-memory reactivation. There was a drug-induced reduction in freezing time relative to controls during Test A_2_ when it was given 9 or 12 h after memory reactivation, but not 18 h later, indicating that the period during which the PL cortex PKC is involved in the persistence of a reactivated aversive memory ranges from 6 to 12 h. It is worth mentioning that animals treated with vehicle 18 h after memory reactivation presented less freezing time during Test A_3_ when compared with their reactivation session, suggesting that extinction learning could have occurred in the control group. Interestingly, this difference was not observed in chelerythrine-treated animals. Future studies are guaranteed to check whether PL cortex PKC inhibition affects the process of extinction.

If PKC involvement in memory persistence depends on prior memory reactivation (i.e. a brief exposure to the conditioning context), then one would expect no changes in memory persistence in animals infused intra-PL cortex with chelerythrine 6 h after their exposure to a non-conditioned context. Indeed, no effects of PKC inhibition were observed when memory reactivation was omitted, a result agreeing with those showed that the induction of persistence-associated mechanisms is triggered by memory reactivation^13,14,25,26^.

The role of conventional PKC isoforms has long been investigated in aversive learning paradigms^27–29^. Recently, the focus has been on the potential involvement of certain atypical PKC isoforms, particularly PKMζ, in the persistence of newly acquired and reactivated aversive memories^30–33^. Considering that chelerythrine has an affinity for both atypical and conventional PKC isoforms^7,34,35^, an additional experiment was performed in which the selective PKMζ inhibitor ZIP was infused intra-PL cortex 6 h after memory reactivation. There was a drug-induced reduction in freezing time when compared with controls during both Tests A_2_ and A_3_, which indicates a PKMζ requirement for the persistence of a reactivated memory. This result agrees with those showing that infusing ZIP into other discrete brain regions affects the persistence of various types of newly acquired memories^8,36–38^. Importantly, since chelerythrine and ZIP effects on memory persistence were similar, chelerythrine action was probably mediated, at least in part, by the inhibition of PKMζ activity. Whatever the case, as shown with chelerythrine, ZIP effects required memory reactivation. This result is of particular relevance since PKMζ inhibition in the absence of memory reactivation has been reported to impair its persistence^8,36,39^. The specificity of ZIP has also been challenged, as it was able to impair the LTP maintenance in PKMζ knockout mice^40^, and inhibit the activity of an atypical PKC isoform termed PKCι/λ^5^, which currently has only been associated with early phases of memory consolidation and early LTP^5^. Thus, one could argue that ZIP effects only are partially associated with PKMζ inhibition. In fact, as a compensatory mechanism, knockout mice for PKMζ increased the expression of PKCι/λ, which in turn mediated the process memory persistence^39^. Besides, in our study the animals are not transgenic, making it less probable that ZIP-induced effects depend on mechanisms other than those mediated by PKMζ.

A study reported that ZIP-induced effects depend on the initial test that occurred 1 day after its infusion into the basolateral amygdala when the fear-potentiated startle was used^23^. Here, the intra-PL infusion of ZIP 6 h after memory reactivation reduced the freezing expression when the animals were retested either 1 or 10 days later, indicating that the time elapsed between drug administration and initial testing is not a pivotal factor influencing the ZIP action. Moreover, it was reported that ZIP infused into the insular cortex impaired the taste aversion memory persistence 1 month after its infusion^36^. We investigated the PL cortex ZIP effects on memory persistence by using a protocol of contextual fear conditioning with a familiarization session, in which the contribution of the medial prefrontal cortex to long-term memory is greater than in protocols without pre-exposure to the context to-be-conditioned^41^, as used in those works^23,36^. Thus, differences in protocols and the brain areas where ZIP was infused may account for the mixed findings observed.

It is currently unknown whether reconsolidation and persistence mechanisms overlap in the PL cortex. To start to address this question, chelerythrine was given immediately after memory reactivation. There was a drug-induced reduction in freezing time relative to controls during Test A_1_, suggesting that PKC activity also influences the contextual fear memory reconsolidation. This result agrees with those showing the importance of the PKC activity during memory reconsolidation in other brain regions^28,42^, and the PL cortex contribution to reconsolidate aversive memories^20,21,43,44^. It is worth mentioning that the chelerythrine group also expressed lower freezing levels than respective controls when tested 7 and 21 days later (Tests A_2_ and A_3_), a result in line with studies showing that interventions targeting the reconsolidation are not associated with extinction-related features, such as reinstatement and spontaneous recovery of original fear memory^24,45,46^. Moreover, it was shown that chelerythrine effects on reconsolidation require prior memory reactivation.

The potential ZIP effects on memory reconsolidation were also investigated. There were no drug-induced changes in freezing time relative to controls during Tests A_1_, A_2_ and A_3_ when it was given immediately after memory reactivation, suggesting that inhibiting the PKMζ at this time point in the PL cortex affects neither the reconsolidation nor the persistence of a reactivated memory. It has been proposed that memory retrieval and reactivation mechanisms may be partially inhibited by ZIP. For instance, the AMPA receptor GluR2A subunit trafficking into synapses is induced by memory retrieval and is necessary for memory reconsolidation^47–49^. Since ZIP’s action is correlated with an inhibition of GluR2A trafficking, this drug could have impaired the memory reactivation and, therefore, the reconsolidation process was not sufficiently induced, which in turn prevented the action of ZIP to occur. It has been shown that PKMζ inhibition into CA1 impaired the reconsolidation of spatial memory^50^. Besides, a reconsolidation-induced enhancement of PKMζ activity in the amygdala was related to the maintenance of olfactory fear memory in juvenile rats^51^. It is plausible that differences in paradigms used (a protocol of spatial memory *vs*. fear memory) may account for the mixed findings reported. Indeed, it has been suggested that PKMζ maintains fear memory in the basolateral amygdala^51^ and the PL cortex^9^, but not in the dorsal hippocampus^52^, although this issue is still under debate. Further, the animals’ age may also influence the outcome since juvenile rats present less memory retention than adult ones^53^. Here, to further address the PKMζ role in memory reconsolidation, another group of rats received ZIP infusion into the PL cortex 1 h after memory reactivation. As depicted in Fig. 5D, one day later, no differences in freezing behavior were observed in ZIP-treated animals relative to controls, suggesting that at this time point there are no effects of PKMζ inhibition on memory reconsolidation. However, there was a reduction in freezing levels when compared to controls during Tests A_2_ and A_3_, suggesting that PL cortex PKMζ activity mediates aspects specifically related to memory persistence as early as 1 h after memory reactivation. This result is consistent with those from the study by Krawczyk *et al. (2016)*^25^. where the inhibition of ERK1/2 in the dorsal hippocampus 3 h after memory reactivation kept the fear memory intact one day later but impaired it when the animals were retested 7 days later. Remarkably, it was recently shown that memory reactivation induces mechanisms related to both memory reconsolidation and persistence^17,25^.

Together, the chelerythrine-induced effects on memory reconsolidation and the lack of ZIP effects on this memory phase suggest a differential contribution of conventional PKC in memory reconsolidation and atypical PKC, such as PKMζ, for memory persistence following reactivation. Future studies could address which PKC isoforms are involved in each memory process in the PL cortex. Altogether, present findings indicate that PL cortex PKC and PKMζ are involved in the reconsolidation and persistence of a reactivated contextual fear memory. Moreover, present findings demonstrated that after the end of the reconsolidation time-window there is an extended opportunity to mitigate the fear memory.

## Material and Methods

### Animals

Adult male Wistar rats weighing 290–320 g (from Biological Sciences Sector of Federal University of Parana) were kept in plastic cages in groups of five per cage with access to food and water *ad libitum*, and maintained on a 12-hour light/dark cycle (lights on at 7:00 am and off at 7:00 pm) and controlled temperature of 22 ± 2 °C. All experiments were performed after the approval of the experimental protocol by the Ethical Committee for the care and use of laboratory animals of the Biological Sciences Sector of Federal University of Parana (authorization number 1011) and were performed in accordance with the Guide for the Care and Use of Laboratory Animals (National Research Council, 2011)^54^.

### Drugs

Chelerythrine (3.0 nmol/0.2 μL/side; Sigma, USA), a selective PKC inhibitor, was dissolved in saline containing 5% of polyoxyethylene sorbitan monooleate (Tween 80), which alone served as vehicle solution. The PKMζ inhibitor ZIP (myristolated PKCζ pseudosubstrate, Anaspec, USA; cat n° AS-63361; 10 nmol/0.2 μl/side) and the scrambled-ZIP (Tocris, USA; cat n° 3215; 10 nmol/0.2 μl/side), were dissolved in phosphate buffer saline (PBS). The dose selection of each drug was based on previously published studies^6,55^.

### Surgery

Rats were anesthetized with ketamine (75 mg/kg; Carlier, Brazil) and xylazine (15 mg/kg; Sespo, Brazil), associated with local anesthesia (3.0% lidocaine with norepinephrine 1:50000; Dentsply, Brazil), and positioned in a stereotaxic frame. After anesthesia, the animals were placed in the stereotaxic frame. Two stainless-steel guide cannulas (length: 11 mm; outer diameter: 0.6 mm) were implanted bilaterally aiming at the PL cortex following the coordinates (AP = + 11.8 mm interaural, ML = ± 0.6 from central suture, DV = − 1.8 from the skull) of the rat brain Atlas of Paxinos and Watson (2009)^56^ and fixed to the skull with two screws and dental acrylic. To avoid possible occlusion, a stylet was introduced inside each guide cannula. Immediately after the surgery, the animals received 0.4 ml of ibuprofen orally (20 mg/ml, Natulab, Brazil). After ten days, the experiments were initiated. At different moments after memory reactivation, the animals received a bilateral infusion with dental needles introduced through the guide cannulas until their tips were 1.5 mm below the cannula end. During 1 min, 0.2 µl/side of either vehicle or drug was injected using two 5.0-µl syringes connected to an infusion pump (Insight, Brazil). A polyethylene catheter was interposed between the upper end of the dental needles and the syringes. The displacement of an air bubble inside the polyethylene was used to monitor drug flow. The needles were removed 45 s after the end of injections.

**Figure 6:**
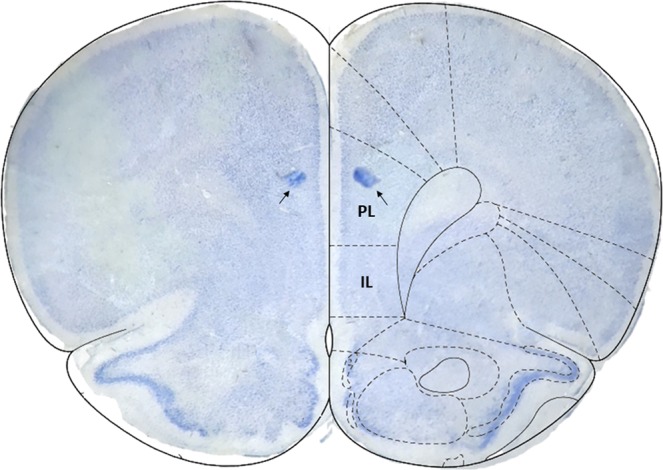
Schematic drawing of the rat medial prefrontal cortex adapted from Paxinos and Watson (2009) atlas highlighting prelimbic (PL) and infralimbic (IL) subregions, and representative infusion site placements (*arrows*) in the PL cortex. Animals were included in the analysis when the treatment was bilaterally infused into the PL cortex.

After the end of experiments, animals were intraperitoneally anesthetized using 1.0 mL/kg of a solution containing xylazine (10 mg/mL, Carlier) and chloral hydrate (2.3 mg/mL, Vetec) intraperitoneally (i.p.). Evans Blue (0.2 µl/ hemisphere) was injected through the guide cannulas for the subsequent evaluation of the locations where vehicle, chelerythrine, ZIP or scrambled-ZIP was infused. Soon after, the brain was removed and immersed in a 10% formalin solution. Brain slices (50 µm thick) were obtained in a vibratome (Leica), mounted on glass microscope slides, and the site of injection was determined. Animals were included in the analysis when both sides of the PL cortex were tagged by Evans Blue (Fig. 6).

### Apparatus

Contextual fear conditioning was performed in a chamber (Context A; 26 ×31.5 ×21 cm; Insight, Brazil), with sidewalls made of aluminum, the front wall and top cover made of transparent acrylic. The floor was made of stainless steel bars (3 mm in diameter and spaced 0.9 mm) connected to a shock-generating font (Insight, Brazil). A neutral chamber (Context B; 34 ×26 ×33 cm) with transparent plexiglass walls and a black cover to provide contextual cues as different as possible from those of Context A was used. Context B was used to assess fear generalization or as a context unable to induce memory reactivation.

### General procedures

The experiments were conducted similarly to previous studies^14,57^, and were performed between 1:00 and 5:00 PM to minimize possible circadian influences on learning and memory processing. All animals were acclimated to the experimenter and the experimental room for 30 min before each session. The experimental rooms were kept under controlled temperature (22 ± 2 °C) and brightness (~ 80 lux). The contextual fear conditioning consisted of the following sessions: on day 1 the animals were placed in Context A for 3 min, where they were allowed to explore it freely and then returned to their home cages. After 24 h, the animals were submitted to the conditioning session in Context A. After the initial 30 s, the animals received three footshocks (0.8 mA/3 s, with an inter-shock interval of 30 s), the unconditioned stimulus (US). After the last shock, the animal remained for an additional 30 s in the conditioning chamber and then returned to the home cage. After 24 h, during the reactivation session, the animals were re-exposed to Context A for 3 min without the US presentation. Immediately, 1, 6, 9, 12 or 18 h after the reactivation session the treatment was bilaterally infused into the PL cortex. After 24 h, the animals were re-exposed to Context A for 3 min (Test A_1_); they were again re-exposed to Context A (3 min) 7 and/or 21 days later (Test A_2_ and Test A_3,_ respectively).

In all experiments, to assess the possible expression of generalized fear, 24 h after Tests A_1_ and A_2_, the animals were exposed to the unpaired and neutral Context B (Test B) for 3 min. Since no fear generalization was observed, this data was omitted. Moreover, Context B exposure was also used to omit memory retrieval. The chambers were cleaned with a 10% ethanol/water solution after each session.

To investigate whether PKC inhibition by chelerythrine interferes with the persistence of a reactivated memory (experiment 1), contextually fear-conditioned animals were randomly allocated to receive a bilateral infusion of vehicle or chelerythrine (3.0 nmol) intra-PL cortex 6, 9, 12 or 18 h after the reactivation session (Context A exposure for 3 min; Fig. 1A).

To investigate whether impairments in memory persistence induced by chelerythrine depends on memory reactivation (experiment 2), contextually fear-conditioned animals were randomly allocated to receive a bilateral infusion of vehicle or chelerythrine (3.0 nmol) intra-PL cortex 6 h after Context B exposure for 3 min (Fig. 2A).

To investigate whether PKMζ inhibition by ZIP interferes with the persistence of a reactivated memory (experiment 3 A), whether impairments in memory persistence induced by ZIP require memory reactivation (experiment 3B), or whether impairments in memory persistence induced by ZIP depend on the initial Test A_1_ (experiment 3 C), contextually fear-conditioned animals were randomly allocated to receive a bilateral infusion of Scr-ZIP (10 nmol) or ZIP (10 nmol) into the PL cortex 6 h after Context A re-exposure or 6 h after Context B exposure for 3 min. The groups were tested after 1 and 7 (Fig. 3A,C) or 10 days later (Fig. 3E).

To investigate whether chelerythrine-induced PKC inhibition also interferes with the reconsolidation of a reactivated memory (experiment 4 A), or whether possible impairments in memory reconsolidation induced by chelerythrine require memory reactivation (experiment 4B), contextually fear-conditioned animals were randomly allocated to receive a bilateral infusion of vehicle or chelerythrine (3.0 nmol) intra-PL immediately after memory reactivation (Fig. 4A) or immediately after Context B exposure for 3 min (Fig. 4C).

To investigate whether ZIP-induced PKMζ inhibition also interferes with the reconsolidation of a reactivated memory (experiment 5), contextually fear-conditioned animals were randomly allocated to receive a bilateral infusion of Scr-ZIP (10 nmol) or ZIP (10 nmol) intra-PL 0 (Fig. 5A) or 1 h (Fig. 5C) after memory reactivation.

Freezing behavior, defined as the total absence of body and head movements except for those associated with breathing^58^, was used as an index of fear memory. Animal behavior was recorded, and freezing time was quantified in seconds by a trained observer blind to the experimental groups and expressed as the percentage of total session time.

### Statistical analysis

The results are expressed as mean ± S.E.M. The percentages of freezing time in the no reactivation (Context B), the reactivation session, and Tests A_1_, A_2_, and A_3_ were submitted to a mixed analysis of variance (ANOVA). The factors evaluated were the treatment and sessions (exposures to Context A and/or Context B). The interaction between treatment and sessions was also assessed. The statistical significance level was set at *P* < 0.05. The Tukey’s test was used for *post-hoc* comparisons when F values achieved statistical significance. GraphPad Prism 8.3 (GraphPad Prism, EUA) was used for statistical analysis and graphing.

The *a priori* sample size determined by power analysis was of eight animals per group (α = 0.05; β = 0.80 and standardized effect size or Cohen’s d = 1.0). The group sizes were equal by design, but due to experimental losses (e.g. when treatment was infused outside the target brain region), in a few cases, they were slightly unequal.

The effect size was calculated using the formula for Hedges’ *g* to reflect the mean differences between two groups (n ≤ 20 per group) that could be dissimilar in size. A *g* ≥ 0.8 was considered a large effect size^59^.

## Data Availability

All data that support this study are available from the corresponding author upon request.

## References

[1] Jaken S (1996). Protein kinase C isozymes and substrates. Curr. Opin. Cell Biol..

[2] Giese KP, Mizuno K (2013). The roles of protein kinases in learning and memory. Learn. Mem..

[3] Ko HG (2016). The role of nuclear PKMζ in memory maintenance. Neurobiol. Learn. Mem..

[4] Gao PP, Goodman JH, Sacktor TC, Francis JT (2018). Persistent increases of PKMζ in sensorimotor cortex maintain procedural long-term memory storage. iScience..

[5] Sacktor, T. C. & Hell, J. W. The genetics of PKMζ and memory maintenance. *Sci Signal*. **10**, 10.1126/scisignal.aao2327 (2017). 10.1126/scisignal.aao2327 PMC6171341 29138296

[6] Pastalkova E (2006). Storage of spatial information by the maintenance mechanism of LTP. Science..

[7] Serrano P (2008). PKMzeta maintains spatial, instrumental, and classically conditioned long-term memories. PLoS Biol..

[8] Li Q (2011). Post-training intra-basolateral amygdala infusions of norepinephrine block sevoflurane-induced impairment of memory consolidation and activity-regulated cytoskeletal protein expression inhibition in rat hippocampus. Neurobiol. Learn. Mem..

[9] Xue YX (2015). Overexpression of protein kinase Mζ in the prelimbic cortex enhances the formation of long-term fear memory. Neuropsychopharmacology..

[10] Crary JF, Shao CY, Mirra SS, Hernandez AI, Sacktor TC (2006). Atypical protein kinase C in neurodegenerative disease I: PKMzeta aggregates with limbic neurofibrillary tangles and AMPA receptors in Alzheimer disease. J. Neuropathol. Exp. Neurol..

[11] Chen C (2016). Epigenetic modification of PKMζ rescues aging-related cognitive impairment. Sci. Rep..

[12] Nakayama D (2015). Long-delayed expression of the immediate early gene Arc/Arg3.1 refines neuronal circuits to perpetuate fear memory. J. Neurosci..

[13] Nakayama D, Yamasaki Y, Matsuki N, Nomura H (2013). Post-retrieval late process contributes to persistence of reactivated fear memory. Learn. Mem..

[14] da Silva TR, Takahashi RN, Bertoglio LJ, Andreatini R, Stern CA (2016). Evidence for an expanded time-window to mitigate a reactivated fear memory by tamoxifen. Eur. neuropsychopharmacol..

[15] Nader K, Schafe GE, Le Doux JE (2000). Fear memories require protein synthesis in the amygdala for reconsolidation after retrieval. Nature..

[16] Przybyslawski J, Sara SJ (1997). Reconsolidation of memory after its reactivation. Behav. Brain Res..

[17] Krawczyk MC, Millan J, Blake MG, Feld M, Boccia MM (2019). Relevance of ERK1/2 post-retrieval participation on memory processes: Insights in their particular role on reconsolidation and persistence of memories. Front. Mol. Neurosci..

[18] Katche C (2010). Delayed wave of c-Fos expression in the dorsal hippocampus involved specifically in persistence of long-term memory storage. Proc. Natl Acad. Sci. USA.

[19] Bekinschtein P (2010). Persistence of long-term memory storage: new insights into its molecular signatures in the hippocampus and related structures. Neurotox. Res..

[20] Stern CA, Gazarini L, Vanvossen AC, Hames MS, Bertoglio LJ (2014). Activity in prelimbic cortex subserves fear memory reconsolidation over time. Learn. Mem..

[21] Vanvossen AC (2017). Newly acquired and reactivated contextual fear memories are more intense and prone to generalize after activation of prelimbic cortex NMDA receptors. Neurobiol. Learn. Mem..

[22] Naseem, M., Tabassum. H. & Parvez, S. PKM-ζ expression is important in consolidation of memory in prelimbic cortex formed by the process of behavioral tagging. *Neuroscience*. 10.1016/j.neuroscience.2019.03.060 (2019). 10.1016/j.neuroscience.2019.03.060 31026567

[23] Parsons RG, Davis M (2011). Temporary disruption of fear-potentiated startle following PKMζ inhibition in the amygdala. Nat. Neurosci..

[24] Stern CA, Gazarini L, Takahashi RN, Guimarães FS, Bertoglio LJ (2012). On disruption of fear memory by reconsolidation blockade: evidence from cannabidiol treatment. Neuropsychopharmacology..

[25] Krawczyk MC (2016). Reconsolidation-induced memory persistence: Participation of late phase hippocampal ERK activation. Neurobiol. Learn. Mem..

[26] Nakayama D, Hashikawa-Yamasaki Y, Ikegaya Y, Matsuki N, Nomura H (2016). Late Arc/Arg3.1 expression in the basolateral amygdala is essential for persistence of newly-acquired and reactivated contextual fear memories. Sci. Rep..

[27] Abeliovich A (1993). PKC gamma mutant mice exhibit mild deficits in spatial and contextual learning. Cell..

[28] Weeber EJ (2000). A role for the beta isoform of protein kinase C in fear conditioning. J. Neurosci..

[29] Bonini JS (2007). On the participation of hippocampal PKC in acquisition, consolidation and reconsolidation of spatial memory. Neuroscience..

[30] Sacktor TC (1993). Persistent activation of the zeta isoform of protein kinase C in the maintenance of long-term potentiation. Proc. Natl Acad. Sci. USA.

[31] Drier EA (2002). Memory enhancement and formation by atypical PKM activity in Drosophila melanogaster. Nat. Neurosci..

[32] Hernandez AI (2003). Protein kinase M zeta synthesis from a brain mRNA encoding an independent protein kinase C zeta catalytic domain. Implications for the molecular mechanism of memory. J. Biol. Chem..

[33] Cai D, Chen S, Glanzman DL (2011). Protein kinase M maintains long-term sensitization and long-term facilitation in aplysia. J. Neurosci..

[34] Ling DS (2002). Protein kinase Mzeta is necessary and sufficient for LTP maintenance. Nat. Neurosci..

[35] Ringvold HC, Khalil RA (2017). Protein kinase C as regulator of vascular smooth muscle function and potential target in vascular disorders. Adv. Pharmacol..

[36] Shema R, Sacktor TC, Dudai Y (2007). Rapid erasure of long-term memory associations in the cortex by an inhibitor of PKM zeta. Science..

[37] Sacktor TC (2011). How does PKMζ maintain long-term memory?. Nat. Rev. Neurosci..

[38] Pauli WM, Clark AD, Guenther HJ, O’Reilly RC, Rudy JW (2012). Inhibiting PKMζ reveals dorsal lateral and dorsal medial striatum store the different memories needed to support adaptive behavior. Learn. Mem..

[39] Zuzina AB, Vinarskaya AK, Balaban PM (2019). Increase in serotonin precursor levels reinstates the context memory during reconsolidation. Invert. Neurosci..

[40] Tsokas, P. *et al*. Compensation for PKMζ in long-term potentiation and spatial long-term memory in mutant mice. Elife. **5**, 10.7554/eLife.14846 (2016). 10.7554/eLife.14846 PMC4869915 27187150

[41] Heroux NA (2018). Differential expression of the immediate early genes c-Fos, Arc, Egr-1, and Npas4 during long-term memory formation in the context preexposure facilitation effect (CPFE). Neurobiol. Learn. Mem..

[42] Girardi BA (2015). Spermidine-induced improvement of reconsolidation of memory involves calcium-dependent protein kinase in rats. Learn. Mem..

[43] Do Monte FH, Souza RR, Wong TT, Carobrez AP (2013). Systemic or intra-prelimbic cortex infusion of prazosin impairs fear memory reconsolidation. Behav. Brain Res..

[44] Levin N, Kritman M, Maroun M, Akirav I (2017). Differential roles of the infralimbic and prelimbic areas of the prefrontal cortex in reconsolidation of a traumatic memory. Eur. Neuropsychopharmacol..

[45] Duvarci S, Nader K (2004). Characterization of fear memory reconsolidation. J. Neurosci..

[46] Asthana, M. K. *et al*. Preventing the return of fear using reconsolidation update mechanisms depends on the Met-Allele of the brain derived neurotrophic factor Val66Met polymorphism. *Int J Neuropsychopharmacol*. **19**, 10.1093/ijnp/pyv137 (2016). 10.1093/ijnp/pyv137 PMC4926796 26721948

[47] Ferrara NC (2019). GluR2 endocytosis-dependent protein degradation in the amygdala mediates memory updating. Sci. Rep..

[48] Jarome TJ (2012). The timing of multiple retrieval events can alter GluR1 phosphorylation and the requirement for protein synthesis in fear memory reconsolidation. Learn. Mem..

[49] Lopez J, Gamache K, Schneider R, Nader K (2015). Memory retrieval requires ongoing protein synthesis and NMDA receptor activity-mediated AMPA receptor trafficking. J. Neurosci..

[50] Rossato JI (2019). PKMζ Inhibition disrupts reconsolidation and erases object recognition memory. J. Neurosci..

[51] Oliver CF (2016). Repeated recall and PKMζ maintain fear memories in juvenile rats. Learn. Mem..

[52] Kwapis JL, Jarome TJ, Lonergan ME, Helmstetter FJ (2009). Protein Kinase Mzeta maintains fear memory in the amygdala but not in the hippocampus. Behav. Neurosci..

[53] Zanca, R. M. *et al*. Contextual fear memory modulates PSD95 phosphorylation, AMPAr subunits, PKMζ and PI3K differentially between adult and juvenile rats. *Neurobiol. Stress* **10**, (2019). 10.1016/j.ynstr.2018.11.002 PMC6430186 30937346

[54] National Research Council. Guide for the care and use of laboratory animals. The National Academies Press. (2011). 21595115

[55] Shi J, Fu LB, Yu LC (2011). Involvement of protein kinase C in the galanin-induced antinociception in the brain of rats. Neurosci. Lett..

[56] Paxinos, G. & Watson, C. The Rat Brain in Stereotaxic Coordinates, Compact sixth ed. *Academic Press*, San Diego. (2009).

[57] Stern CA (2015). Δ9-Tetrahydrocannabinol alone and combined with cannabidiol mitigate fear memory through reconsolidation disruption. Eur. Neuropsychopharmacol..

[58] Blanchard RJ, Blanchard DC (1969). Crouching as an index of fear. J. Comp. Physiological Psychology..

[59] Ellis, P. D. The essential guide to effect sizes: statistical power, meta-analysis, and the interpretation of research results. Cambridge University Press. (2010).

